# The emerging role of AMPK in the regulation of breathing and oxygen supply

**DOI:** 10.1042/BCJ20160002

**Published:** 2016-08-30

**Authors:** A. Mark Evans, Amira D. Mahmoud, Javier Moral-Sanz, Sandy Hartmann

**Affiliations:** * Centre for Integrative Physiology, College of Medicine and Veterinary Medicine, Hugh Robson Building, University of Edinburgh, Edinburgh EH8 9XD, U.K.

**Keywords:** AMP-activated protein kinase (AMPK), apnoea, Ca^2+^–calmodulin-activated kinase kinase-β (CaMKK-β), hypoxia, liver kinase B1 (LKB1), pulmonary, ventilation

## Abstract

Regulation of breathing is critical to our capacity to accommodate deficits in oxygen availability and demand during, for example, sleep and ascent to altitude. It is generally accepted that a fall in arterial oxygen increases afferent discharge from the carotid bodies to the brainstem and thus delivers increased ventilatory drive, which restores oxygen supply and protects against hypoventilation and apnoea. However, the precise molecular mechanisms involved remain unclear. We recently identified as critical to this process the AMP-activated protein kinase (AMPK), which is key to the cell-autonomous regulation of metabolic homoeostasis. This observation is significant for many reasons, not least because recent studies suggest that the gene for the AMPK-α1 catalytic subunit has been subjected to natural selection in high-altitude populations. It would appear, therefore, that evolutionary pressures have led to AMPK being utilized to regulate oxygen delivery and thus energy supply to the body in the short, medium and longer term. Contrary to current consensus, however, our findings suggest that AMPK regulates ventilation at the level of the caudal brainstem, even when afferent input responses from the carotid body are normal. We therefore hypothesize that AMPK integrates local hypoxic stress at defined loci within the brainstem respiratory network with an index of peripheral hypoxic status, namely afferent chemosensory inputs. Allied to this, AMPK is critical to the control of hypoxic pulmonary vasoconstriction and thus ventilation–perfusion matching at the lungs and may also determine oxygen supply to the foetus by, for example, modulating utero-placental blood flow.

## INTRODUCTION

Regulated oxygen supply is key to the maintenance of oxidative phosphorylation and thus cellular energy status in mammals, not least because of the limited capacity for cellular oxygen storage relative to the extensive reserves of other substrates. It was proposed, therefore, that natural selection may have employed AMP-activated protein kinase (AMPK) to co-ordinate system-level adjustments of whole-body function in response to oxygen deficits in animals [1]. Consistent with this view, recent studies on high-altitude Andean populations have shown that the gene for the AMPK-α1 subunit (*PRKAA1*) has been influenced by natural selection through single nucleotide polymorphisms [2]. Confirmation of a role for AMPK in oxygen delivery has now been provided by conclusive experimental evidence that, in addition to its well-recognized capacity as a regulator of cell-autonomous pathways of energy supply [3], AMPK is essential to the regulation of breathing during hypoxia and thus oxygen and energy distribution to the body [4].

## FRAGMENTS FROM THE LIBRARIES OF BABYLON

### The AMP-activated protein kinase

AMPK is a cellular energy sensor that acts to maintain energy homoeostasis. It exists as heterotrimers comprising one of two catalytic α subunits, in combination with one each of the two β and three γ regulatory subunits, which together may form at least 12 different heterotrimeric subunit combinations [5,6]. In this respect it is important to note that evidence is now emerging to suggest that different subunit combinations may be selected by a given cell type, that each combination may exhibit different sensitivities to activation by AMP and ADP and thus metabolic stresses, and that each may selectively phosphorylate and regulate a different spectrum of target proteins [8].

**Figure 1:**
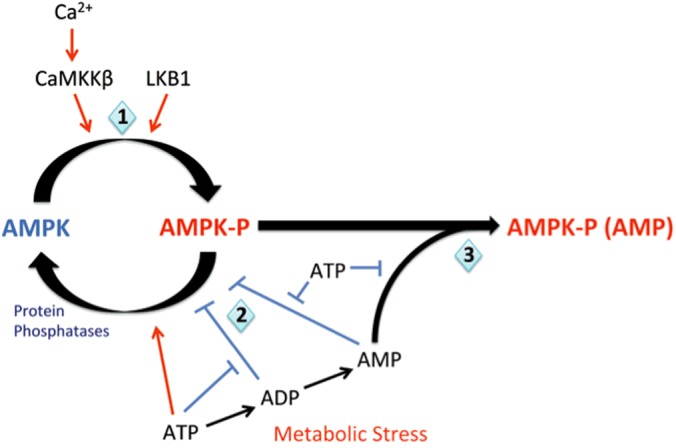
Regulation of the AMP-activated protein kinase (1) AMPK is constitutively phosphorylated (AMPK-P) by LKB1. However when ATP is bound to AMPK, dephosphorylation by protein phosphatase 2C (PP2C) is promoted and AMPK remains deactivated (AMPK). Metabolic stresses, such as hypoxia, increase the AM(D)P/ATP ratio and promote displacement of ATP by AMP, and to a lesser extent by ADP, from three sites on the AMPK γ subunit. Binding of AMP or ADP to the γ subunit may promote phosphorylation by LKB1 and at the same time (2) inhibit dephosphorylation by PP2C. (3) AMP, but not ADP, binding also promotes further allosteric activation of AMPK. These three mechanisms deliver AMPK activation in response to metabolic stresses. In addition, AMPK can be activated in a Ca^2+^-dependent manner through CaMKK-β, which phosphorylates the same γ subunit site as LKB1. Figure adapted from [179]: Hardie, D.G., Salt, I.P., Hawley, S.A. and Davies, S.P. (1999) AMP-activated protein kinase: an ultrasensitive system for monitoring cellular energy charge. Biochem. J. **338**, 717–722.

AMPK activities are exquisitely coupled to mitochondrial metabolism through changes in the cellular AMP/ATP and ADP/ATP ratios (Figure 1). There are four nucleotide-binding sites (CBS repeats) on the γ subunit, of which only sites designated 1, 3 and 4 may ever be occupied [8]. Binding of AMP to the γ subunit causes a 10-fold increase in AMPK activity by allosteric activation, with further activation of up to 100-fold generated by binding of either AMP or ADP through their promotion of phosphorylation and inhibition of dephosphorylation at Thr^172^ on the α subunit; each of these effects is opposed by ATP [9,10]. Thr^172^ is primarily phosphorylated by the tumour-suppressor kinase liver kinase B1 (LKB1), which appears to be constitutively active but phosphorylates AMPK more rapidly when AM(D)P is bound to the γ subunit [11]. There is also an alternative Ca^2+^-dependent activation mechanism, the calmodulin-dependent protein kinase Ca^2+^–calmodulin-activated kinase kinase-β (CaMKK-β), which phosphorylates Thr^172^ and thus activates AMPK in an AMP-independent manner [5,6,12]. Contrary to previous proposals [13], however, there is little evidence to support the view that AMPK is directly activated by reactive oxygen species (ROS) [14,15]. Once activated the classical action of AMPK is to phosphorylate targets that switch off non-essential anabolic processes that consume ATP and switch on catabolic pathways that generate ATP [12], thereby compensating for deficits in ATP supply via, for example, reductions in mitochondrial oxidative-phosphorylation.

Intriguingly, in the context of the present discussion, the genes encoding the α and γ subunits of the AMPK orthologue of yeast *Saccharomyces cerevisiae* (*SNF1* and *SNF4*) support colony-level metabolic adaptation [16–19]. For example, in a high glucose environment, yeast initially grow rapidly using glycolytic metabolism to generate ATP, but when glucose runs low the growth rate decreases as yeast undergo diauxic shift towards greater reliance on mitochondrial oxidative phosphorylation. This adaptation to deficits in substrate supply is blocked in yeast with *snf1* or *snf4* mutations that are unable to support the diauxic shift [16,17,20]; i.e. they can only grow on a source of glucose.

In an evolutionary context, this observation raised the possibility that natural selection may have deployed AMPK to govern the adaptation of animals to deficits in oxygen and thus energy supply at both the cellular and whole-body level. Moreover, the fact that AMPK is a serine/threonine kinase suggested the capacity for regulation of processes outside of metabolism such as ion channel activity, which our findings [21–24] and those of others have since confirmed. For example, AMPK may phosphorylate and ‘inactivate’ the pore-forming α subunit of multiple calcium-activated potassium channels (K_Ca_1.1 and K_Ca_3.1) [22,25], the voltage-gated potassium channel Kv1.5 [24,26,27] and the ATP-inhibited K_ATP_ channel (Kir6.2) [28], or may phosphorylate and ‘activate’ the α subunit of the voltage-gated potassium channel Kv2.1 [21]. AMPK has the potential to thus increase or decrease cell excitability, in a manner determined by the cell-specific expression of members of the ion channel superfamily, and thereby deliver system-level control of whole-body metabolic status [1].

We have now provided conclusive evidence that the LKB1/AMPK signalling pathway does indeed play a critical role in modulating the delivery of oxygen to the body [4,29], in addition to its well-recognized role in regulating cell-autonomous pathways of energy supply [3]. Perhaps most significantly, our data suggest that LKB1/AMPK signalling pathways act not only to optimize ventilation during hypoxia, but also to oppose respiratory depression during hypoxia and may thus resist hypoventilation and apnoea [4]. However, the locus at which AMPK co-ordinates the hypoxic ventilatory response was not as one would have predicted.

### Regulation of rhythmic ventilation

**Figure 2:**
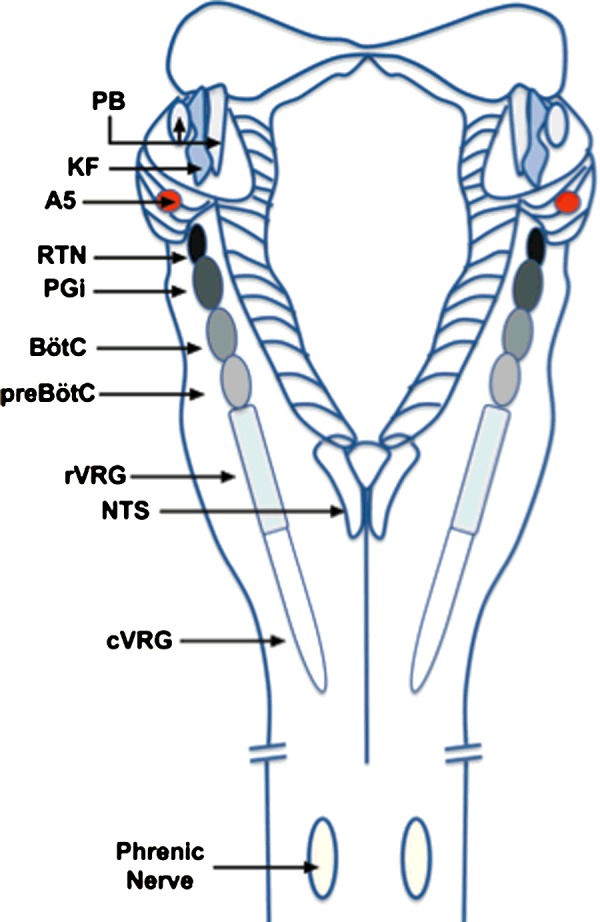
Functional compartments of the brainstem ventilatory respiratory columns Dorsal view of the brainstem illustrating the functional compartments within the ventilatory respiratory column. KF, Kölliker-Fuse nucleus; PB, parabrachial nuclei; NA, noradrenergic A5 area; RTN, retrotrapezoid nucleus; PGi, paragigantocellular reticular nucleus; BötC, Bötzinger complex; preBotC, pre-Bötzinger complex; rVRG, rostral ventral respiratory group; cVRG, caudal ventral respiratory group. Image adapted from [180]: Rekling, J.C. and Feldman, J.L. (1998) PreBotzinger complex and pacemaker neurons: hypothesized site and kernel for respiratory rhythm generation. Annu. Rev. Physiol. **60**, 385–405.

**Figure 3:**
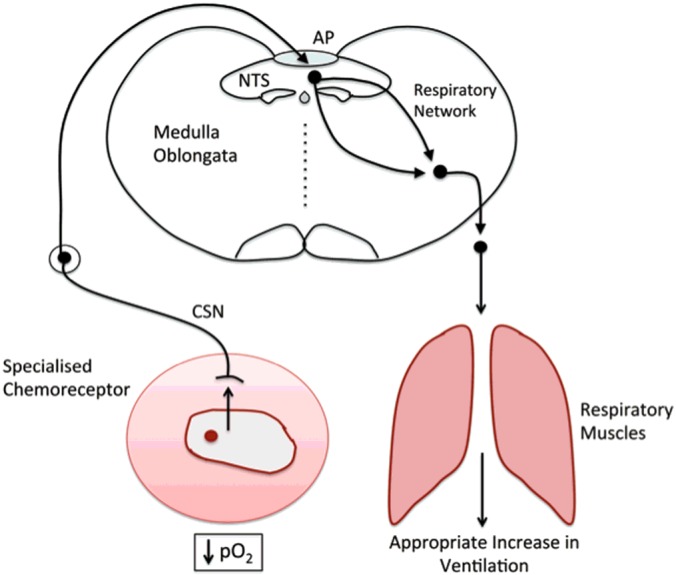
The hypoxia-responsive respiratory network from carotid body to brainstem The hypoxia-responsive respiratory network spans the catecholaminergic cells of the carotid body type I cells, dorsal A2, C2 and ventral A1 and C1 neurons of the caudal brainstem, which are located at the AP, NTS and the ventrolateral medulla. The respiratory central pattern generators comprise: RTN, retrotrapezoid nucleus; BötC, Bötzinger complex; preBotC, pre-Bötzinger complex; rVRG, rostral ventral respiratory group; cVRG, caudal ventral respiratory group.

That ventilatory adjustments are critical to the body's capacity to accommodate variations in oxygen demand and supply during sleep and ascent to altitude is exemplified by the fact that adaptation of mammals to hypoxia at altitude is initially characterized by progressive increases in ventilatory drive, which partially restore arterial *P*O_2_ and protect against apnoea [30]. Ventilatory movements are delivered by motor neuronal pathways that are informed by respiratory central pattern generators (rCPGs), which are distributed bilaterally in the pons and ventral medulla of the brainstem (Figure 2) [31]. These semi-autonomous neural networks comprise core circuits of excitatory and inhibitory interneurons that deliver rhythmic patterns of activity [32], and confer a set-point about which respiratory rhythm is continuously modulated through the integration of inputs from those central [32,33] and peripheral chemosensors [34] which monitor oxygen, carbon dioxide and pH. It is generally accepted that the carotid bodies, which reside at the bifurcation of the common carotid artery, represent the primary peripheral chemoreceptors [34] and that the acute hypoxic ventilatory response is delivered by increased afferent discharge from the carotid bodies to the rCPGs via, in great part, catecholaminergic networks within the caudal brainstem (Figure 3) [35,36].

To assess the role of LKB1 and AMPK in this process, we used the tyrosine hydroxylase promoter to drive deletion of *AMPK-α1* and *-α2* genes in all catecholaminergic cells [4], including therein type I cells of the carotid and aortic bodies [34,37], and downstream neurons within the brainstem respiratory network that relay afferent inputs to the rCPGs [38]. Both *LKB1* and *AMPK* deletion precipitated pronounced ventilatory dysfunction during hypoxia [4,29] that was characterized by marked attenuation of the hypoxic ventilatory response, and which ultimately led to hypoventilation rather than hyperventilation and frequent prolonged apnoeas.

Upon hypoxia at altitude or during sleep, activation of LKB1/AMPK signalling pathways may therefore aid appropriate ventilatory adjustments and thus ‘protect’ against acute ventilatory instability [30], although deficiency of either may confer greater susceptibility to disordered breathing. In this respect it is notable that, of the two available α subunits, selective loss of the *AMPK-α1* catalytic subunit was the primary precipitant of ventilatory dysfunction during hypoxia [4]; consistent with the finding that natural selection in high-altitude (Andean) populations has led to single nucleotide polymorphisms in *PRKAA1* [2].

Given that it is widely accepted that the carotid bodies drive the entire ventilatory response to a fall in arterial *P*O_2_, we had always presumed that this organ would be the primary site of AMPK action in this respect. Not for the first time, however, the order in which evolution may have influenced the development and thus organization of body systems appears, it now seems, counterintuitive.

## A PINCH OF PTOLEMY–AMPK AND THE CAROTID BODY

The carotid bodies were identified as sensory organs by De Castro in 1928 [39], after which Heymans and Bouckaert [40] established that they mediated hyperventilation in response to a fall in arterial *P*O_2_ and thus defined these organs as the primary peripheral arterial chemoreceptors. The carotid body type I (glomus) cells underpin chemosensory activity [41], when upon exposure to hypoxia and/or hypercapnia they release a variety of neurotransmitters which elicit increases in afferent fibre discharge along the carotid sinus nerve and thereby govern cardiorespiratory reflexes that elicit corrective changes in ventilation [42–45]. Recent evidence now suggests that the aortic bodies, which are located at the aortic arch, are similarly activated during hypoxia and/or hypercapnia and may also contribute to the hypoxic ventilatory response [37]. Type I cells of the carotid and aortic bodies therefore define a class of oxygen-sensing cells, in which the *P*O_2_ at which mitochondrial oxidative phosphorylation is inhibited during hypoxia (≤60 mmHg oxygen) is higher than in other cell types [46–48]. Once this threshold is breached hypoxia-induced changes in cell activity increase in a manner related to the degree of hypoxia [46,49], that is until these activities begin to fail under near anoxic conditions (<2% oxygen) [50]; at this point mitochondrial oxidative phosphorylation is inhibited in cells that do not function to monitor oxygen supply [47]. In short, all oxygen-sensing cells function to respond to deficits in oxygen supply over the physiological range of *P*O_2_.

### Mitochondria underpin hypoxia-response coupling in carotid body type I cells

A significant body of evidence now argues in favour of the view that type I cell activation during hypoxia is consequent to the inhibition of mitochondrial function. In retrospect the initial clue to this fact was provided by the seminal work of Heymans and Bouckaert [40], in that they demonstrated that cyanide mimicked and occluded the activation by hypoxia of the carotid body. However, the first direct evidence was obtained through the analysis of the respiratory chain redox status [51]. By relating outcomes to afferent sinus nerve discharge during hypoxia, it was shown that an increase in the NAD(P)H/NAD(P)^+^ ratio correlated with changes in afferent nerve activity over the physiological range of oxygen levels. At the time it was proposed that mitochondria of most cells may utilize a high-affinity (i.e. normal) cytochrome *a*_3_, whereas the cytochrome *a*_3_ incorporated in mitochondria of oxygen-sensing cells may have a low affinity for oxygen. Consistent with this hypothesis, recent investigations have demonstrated that NDUFA4L2 [52] and COX4I2 [53,54], two nuclear-encoded atypical subunits of the mitochondrial electron transport chain, are constitutively expressed in carotid body type I cells under normoxia [55]. This contrasts with a number of other cell types where NDUFA4L2 and COX4I2 expression is ordinarily low, but is increased during prolonged hypoxia [52–54]. Both NDUFA4L2 and COX4I2 reduce the capacity for mitochondrial oxygen consumption and act to limit mitochondrial ROS production during hypoxia, by reducing the activity of complex I and cytochrome *c* oxidase respectively. In this respect it is interesting to note that allosteric modulation of cytochrome *c* oxidase (COX) is delivered by COX4 in a subtype-specific manner, with COX4I1 but not COX4I2 conferring COX inhibition by ATP [54,56], i.e. in carotid body type I cells it seems unlikely that the rate of oxygen consumption and thus ATP supply via mitochondrial oxidative phosphorylation will increase during hypoxia as ATP levels fall [53,56–58]. It has been suggested, therefore, that constitutive expression of NDUFA4L2 and COX4I2 by carotid body type I cells might determine the affinity of their mitochondria for oxygen and thus confer, in part, the capacity of these cells to monitor changes in arterial oxygen supply. Intriguingly, COX4I2 is also constitutively expressed by pulmonary arterial myocytes [58,59] and neurons of the central nervous system [56], which may in some instances also function to monitor oxygen supply (see below).

That mitochondria may be the site of oxygen-sensing within type I cells of the carotid body is supported by the fact that, in addition to cyanide, all inhibitors and uncouplers of mitochondrial electron transport both mimic and occlude the effects of hypoxia [60]. Moreover, recent studies have shown that conditional deletion in type I cells of *Ndufs2*, a mitochondrial complex I gene that participates in ubiquinone binding, blocks carotid body activation during hypoxia [61].

### ATP, LKB1, AMPK and hypoxia-response coupling in carotid body type I cells

What remains open to debate is the precise nature of the signalling pathway(s) which couples inhibition by hypoxia of mitochondrial oxidative phosphorylation to the activation of oxygen-sensing cells, such as type I cells, and whether or not all oxygen-sensing cells utilize a common signalling pathway. At the very least one would expect an initial fall in ATP supply and associated ADP accumulation that would be compensated for, in the immediate term, by the adenylate kinase reaction, leading to consequent increases in the AMP/ATP ratio [62,63]. When one considers this and the fact that AMPK is intimately coupled to mitochondrial metabolism via both increases in the AM(D)P/ATP ratio and LKB1, the possibility that AMPK may contribute to hypoxia-response coupling is immediately apparent. If this were the case, then one would naturally expect any contribution of AMPK to ventilatory control to be delivered at the level of the carotid body type I cell and through the consequent inhibition of those ‘oxygen-sensing’ potassium channels known to underpin their chemosensory response. Not least because thereafter the generally held viewpoint is that carotid body afferent inputs to the brainstem activate subordinate relays that modulate rCPG activities and thus increase ventilation.

Our preliminary investigations into the role of the LKB1/AMPK signalling pathway appeared entirely consistent with this view, in that conditional deletion of *LKB1* virtually abolished the capacity for type I cell activation during hypoxia, increases in afferent discharge and, like *AMPK* deletion, attenuated the hypoxic ventilatory response [64,65]. Contrary to these findings and against our expectations, however, *AMPK* deletion failed to attenuate afferent discharge from the carotid body, yet caused even greater attenuation of the hypoxic ventilatory response [4] when compared with *LKB1* deletion (unpublished work). This runs counter to our previous pharmacological studies, which suggested that 5-amino-4-imidazolecarboxamide riboside (AICAR), an AMPK agonist [66], activated carotid body type I cells and increased afferent discharge [67], and that this action was inhibited by the AMPK antagonist compound C. However, compound C is a very non-selective kinase inhibitor, which in a screen of 70 protein kinases was shown to inhibit at least ten other kinases more potently than AMPK [68]. Moreover, off-target effects of other pharmacological tools have also been identified, such as inhibition by AICAR of adenosine transporters [69] (adenosine receptors being key modulators of type I cell activity [70]) and/or AICAR-mediated reductions in the adenylate pool and ATP [71,72]. One must therefore conclude that AMPK is not *necessary* for type I cell activation by hypoxia. Consistent with this view, recent studies on the actions of two different AMPK activators, AICAR and A769662 [73], suggest that these agents neither precisely mimic the effects of hypoxia on nor induce pronounced activation of carotid body type I cells [74,75], and our own most recent investigations now support this view (unpublished work).

Nevertheless it would appear that we have inadvertently uncovered a split in the dependency on LKB1 and AMPK respectively of carotid body activation during hypoxia on the one hand and the hypoxic ventilatory response on the other. The reasons for this remain to be resolved, but experimental outcomes perhaps point to hierarchical control of the respiratory network by LKB1, AMPK and one or more of the 12 AMPK-related kinases [76]. Given that afferent discharge is, in great part, triggered by exocytotic release of ATP from type I cells [77], it is quite plausible that LKB1 may maintain, in an AMPK-independent manner, the capacity for ATP synthesis and/or exocytosis within type I cells, and thus afferent discharge from the carotid body. This is entirely in keeping with the fact that LKB1 may govern glucose homoeostasis [78,79] and mitochondrial function [80,81] independently of AMPK, perhaps via constitutive phosphorylation of an AMPK-related kinase [76,82,83], given that *LKB1* deletion has been shown to decrease mitochondrial membrane potential and basal ATP levels in other cell types [80,81,84]. It is therefore possible that any cell lacking LKB1, such as carotid body type I cells, may be unable to sustain appropriate cellular energy charge and activity due to defective mitochondrial function, either at rest or during exposure to metabolic stresses such as hypoxia.

So where does this leave us? Well one backward look takes us to ATP, ADP and AMP levels and the inhibition during hypoxia of type I cell K^+^ channels, which ultimately triggers exocytosis [85–87]. The principal players in this respect are the large conductance voltage- and Ca^2+^-activated K^+^ current (BK_Ca_) [88,89] and the voltage-independent TASK-like leak K^+^ current [90–92], although it should be noted that variations in channel expression may confer identified species differences [93,94] and contribute to changes of oxygen sensitivity during postnatal maturation [95,96]. It is now clear that hypoxia (and hypercapnia) principally acts to depolarize type I cells by inhibiting TASK1/3 K^+^ channels [74], leading to Ca^2+^ entry through voltage-gated Ca^2+^ channels, consequent exocytosis and ATP release. Moreover, in the absence of a determining role for AMPK [4,75], substantial evidence now supports the view that TASK K^+^ channels directly monitor the adenylate pool [97], and close when ATP levels fall consequent to the inhibition by hypoxia of mitochondrial oxidative phosphorylation [60]. AMPK does, however, phosphorylate and, like hypoxia, inhibit BK_Ca_ channels of carotid body type I cells [22], the archetypal oxygen-sensing potassium channel [85,89]. This action will clearly have functional consequences with respect to transmitter release, conceivably by modulating the transition to ‘bursting’ patterns of action potential firing [98], but these remain to be resolved.

In short, type I cell activation during hypoxia is probably precipitated by changes in the adenylate pool and ATP [99], and membrane depolarization due to subsequent inhibition of K^+^ currents carried by TASK1/3 heterodimers [91,100]. However, the primacy of this view has recently been challenged by three alternative hypotheses:

1. It has been suggested that type I cell activation may be triggered by increases in hydrogen sulfide production consequent to a fall in carbon monoxide synthesis during hypoxia [101], although the findings of others suggest that activation of carotid body type I cells by exogenous hydrogen sulfide results from direct inhibition of mitochondrial oxidative phosphorylation [102]. The effects on type I cells of hydrogen sulfide may not, therefore, be inconsistent with the conclusion drawn above. This perspective has recently received support from single-cell transcriptome analysis of mouse type I cells which identified few to no reads of the enzymes responsible for generating either carbon monoxide or hydrogen sulfide [55], respectively, haem oxygenase-2, or cystathionine-γ-lyase and cystathionine-β-synthase.
2. As mentioned previously, conditional deletion in tyrosine hydroxylase-positive cells of *Ndufs2*, a mitochondrial complex I gene which encodes a protein that participates in ubiquinone binding, has also been shown to selectively block carotid body activation during hypoxia (but not hypercapnia or hypoglycaemia) and thus the hypoxic ventilatory response [61]. The authors concluded that this probably results from loss, during hypoxia, of the capacity for signalling via increased generation of mitochondrial ROS. However this study did not address the impact of *Ndufs2* deletion on oxidative phosphorylation in type I cells, the capacity for inhibition of type I cell mitochondrial oxidative phosphorylation during hypoxia and consequent modulation of TASK-like potassium currents by alterations in the adenylate pool (see also [103]). Furthermore, and as discussed above, NDUFA4L2 and COX4I2 are constitutively expressed by type I cells and act to limit mitochondrial ROS production during hypoxia [53,54]. That aside, it is important to note that conditional deletion of *Ndufs2* in catecholaminergic cells blocked the hypoxic ventilatory response even though the capacity for both basal and activated transmitter release was retained by type I cells (see below for further discussion).
3. Most recently a novel chemosensory signalling pathway has been proposed to be a prerequisite for type I cell activation during hypoxia, namely lactate-dependent activation of olfactory receptor 78 (Olfr78) [104]. In this study global deletion of *Olfr78* was found to block carotid body activation during hypoxia and thus the hypoxic ventilatory response of mice. By virtue of a requirement for lactate production and release consequently to induction of anaerobic glycolysis, the proposed model for lactate-dependent activation of Olfr78 during hypoxia is consistent with the mitochondrial hypothesis, but is inconsistent with a mechanism in which type I cell activation is determined by TASK K^+^ channel inhibition through alterations in the adenylate pool [74]. That is unless, of course, these two pathways converge. Once again, however, it may be worthy of note that the hypoxic ventilatory response was blocked by global *Olfr78* deletion despite the fact that basal afferent discharge from the carotid body was retained (see below for further discussion).

Putting due scrutiny of the aforementioned signalling pathways to one side, it is clear from our own findings that all pathways key to carotid body type I cell activation during hypoxia must be, in some way, dependent on the continued expression of LKB1, but not AMPK, and a sufficiency of mitochondrial function and/or ATP supply.

So how can it be that both *LKB1* and *AMPK* deletion block the hypoxic ventilatory response, when deletion of the latter does not adversely affect carotid body activation during hypoxia [4,29,64]? For such a proposal runs contrary to the generally held view that increased afferent discharge from carotid body to brainstem determines the ventilatory response to a fall in arterial *P*O_2_ [34]. Well there is substantial evidence to support an alternative yet inclusive perspective, namely that the hypoxic ventilatory response is determined by the co-ordinated action of the carotid body and a hypoxia-responsive circuit within the brainstem. We will see that this must now be borne in mind when drawing conclusions from all studies described above that employed either global knockout strategies or conditional gene deletion in catecholaminergic cells.

## A DASH OF COPERNICUS–AMPK AND THE BRAIN-CENTRED CHEMOSENSORY NETWORK

From here on in our aim is to be a little more provocative if not heretical, at least in the eyes of some respiratory physiologists, by giving emphasis to a matter that has long been quietly considered by a minority of the field. In actual fact, our investigation is merely the latest in a long line to have described experimental observations that run counter to the standard model for the control of ventilation by peripheral chemosensors, and the pre-eminence of the carotid bodies in this respect.

Not surprisingly, in retrospect, the possibility that peripheral chemosensors may not be the sole arbiters of the hypoxic ventilatory response has been suggested by investigations on the evolution of ventilatory control systems, most notably with respect to the demonstration that oxygen-sensing occurs and a component of the hypoxic ventilatory response arises at the level of the caudal brainstem in amphibians, with both the location and influence of the primary peripheral chemosensors changing during the ascent from gill-breathing tadpole to lung-assisted air-breathing adult [105,106]. In fact one could quite reasonably argue that evolutionary pressures have periodically led to the reconfiguration of peripheral chemoreceptor inputs [106] about a common ancestral hypoxia-sensor within the caudal brainstem, that underpins signal integration and thus acts as the ‘gatekeeper’ of respiratory adjustments during hypoxia. That said, the possibility that neural networks within the brainstem of mammals might respond to central hypoxia was first raised over 35 years ago by the work of Dampney and Moon [107], during their investigations on the central ischaemic vasomotor response. Thereafter, during their investigations on Cushing's reflex [108], Sun and Reiss demonstrated that both cyanide and hypoxia activated neurons within the rostral ventrolateral medulla [109,110], mirroring Heymans and Bouckaert's earlier work on the carotid body. Moreover extensive evidence has been provided in support of the view that increases in ventilation are initiated by brainstem hypoxia in the presence of only basal normoxic afferent input from the carotid bodies [111,112], and it has been suggested that different aspects of the brainstem respiratory network may exhibit different sensitivities to hypoxia [113].

To date, however, little emphasis has been placed on the role of hypoxia-sensing at the brainstem, perhaps because the hypoxic ventilatory response is so effectively abolished by resection of the carotid sinus nerve in humans [114]. Yet extensive investigations have demonstrated that following carotid body resection, hypoxia-responsive catecholaminergic neurons of the caudal brainstem may underpin partial recovery of the hypoxic ventilatory response [115], at least in rodents, and it is recognized that loss of these neurons underpins ventilatory dysfunctions associated with Rett syndrome, including hypoventilation and apnoea, which are exacerbated during hypoxia [116].

Consistent with outcomes of the aforementioned studies, our findings strongly suggest that AMPK governs the activation of previously identified hypoxia-responsive nuclei within the caudal brainstem [110,117], and thus supports the delivery of increased respiratory drive during hypoxia that is required to protect against hypoventilation and apnoea. The most convincing evidence of this was provided by examination of brainstem function in *AMPK* knockout mice by functional magnetic resonance imaging (fMRI), which identified reduced activation during hypoxia of discrete dorsal and ventral nuclei of the caudal brainstem, despite the fact that carotid body afferent discharge was retained [4]. This was corroborated by analysis of immediate early gene (c-*fos*) expression.

The caudal location relative to Bregma of the dorsal active region is consistent with areas of the nucleus tractus solitarius (NTS) that are activated by hypoxia and which represent the primary site of receipt of carotid body afferent input [35,117,118]. Here *AMPK* deletion selectively attenuated c-*fos* expression during hypoxia by mixed subpopulations of C2 neurons and A2 neurons (SubP; SolM) within the medial subnucleus proximal to the midline and the area postrema (AP) [4], which have been previously shown to be activated during hypoxia [38]. A2 neurons of the AP/NTS provide afferent input to and determine, together with the carotid body, activation by hypoxia of A1/C1 neurons within the ventrolateral medulla [38,119], the position of which [119] aligns well with the ventral active region identified by fMRI analysis [4]; by contrast projections of the NTS mostly avoid key components of the rCPGS [119], namely the Bötzinger and pre-Bötzinger complexes [120]. Analysis of c-*fos* expression at the level of the ventrolateral medulla suggested that *AMPK* deletion selectively reduced the activation of A1 neurons during hypoxia, although it should be noted that there is significant overlap between the most caudal C1 and the most rostral A1 neurons [121]. Our findings therefore suggest that the hypoxic ventilatory response, including that provided by afferent inputs from peripheral chemosensors, is attenuated by loss of AMPK function at the level of the caudal brainstem, within a neuronal circuit spanning the C2/A2 neurons of the NTS and A1 neurons of the ventrolateral medulla. This is consistent with optogenetic and pharmacological interventions at the level of the NTS [117,122], and the proposal that NTS neurons lie on the sensory side of the central respiratory network [123,124]. We cannot rule out the possibility that suppression of the hypoxic ventilatory response in *AMPK* knockouts may be allied to exacerbation of the Cushing reflex [35,108]. However, this reflex is only elicited under anaesthesia and by ischaemic hypoxia (∼1% O_2_), and is maintained or enhanced by hypercapnia [35,108,125]. By contrast, hypoxic ventilatory depression was evident in conscious *AMPK* knockouts during mild and severe hypoxia, as were deficits in brainstem activity, and was reversed rather than exacerbated by hypercapnia.

Surprisingly, we observed pronounced right–left asymmetry of brainstem activation during hypoxia, which may provide for specialization sufficient to prevent delays in respiratory responses to hypoxic stress by limiting conflicting outputs from each side of the brain [126], as has been proposed previously with respect to cognitive performance [127]. Further investigation will be required to determine how right–left asymmetry may be orchestrated by the complex interplay of neurotransmitters deployed during hypoxia and the role of AMPK in such processes of selection. In this respect it is notable that C2 and A2 neurons are both catecholaminergic and glutamatergic [123,128], and that 6–10% of tyrosine hydroxylase-positive C2, A2 and A1 neurons also express neuronal nitric oxide synthase, which supports the hypoxic ventilatory response by synthesizing NO [129] and/or *S*-nitrosothiols [130], and in a manner that may be facilitated by AMPK [131].

It could be argued that *AMPK* deletion in catecholaminergic cells simply leads to the failure of central integration and transduction of peripheral chemoafferent input and consequent failure of the hypoxic ventilatory response, due to the inability of affected neurons to maintain appropriate levels of activity when exposed to metabolic stress [132]. However, following *AMPK* deletion, carotid body afferent discharge remained exquisitely sensitive to a fall in *P*O_2_ and ventilatory responses to hypercapnia remained unaffected even during severe (8%) hypoxia, which clearly demonstrates that *AMPK* deletion does not compromise the capacity during hypoxia for activation of chemosensory catecholaminergic neurons, exocytosis nor effective delivery of increased respiratory drive. This is consistent with the observation that neuronal integrity during hypoxia may be preserved, in part, by AMPK-independent mechanisms [133] that maintain ATP supply by accelerating glycolysis and in a manner supported by mobilization of astrocyte glycogen stores [134]. If one accepts this position, then AMPK must aid the modulation by hypoxia of discrete nuclei within the caudal brainstem that deliver increased drive to breathe via neural networks that modulate the rCPGs [36], and which may also co-ordinate functional hyperaemia [135].

## THE CASE FOR SIGNAL INTEGRATION AT AN OXYGEN-SENSING NUCLEUS WITHIN THE BRAINSTEM

The phrase ‘to say more would be pure speculation’ is often uttered and rightly so, at least when interpreting experimental outcomes. In the present context, however, we are happy to invite ridicule and scorn for the sake of greater debate and experimental inquisition, and to achieve this goal we bring to centre stage the possibility that a cluster of hypoxia-responsive neurons proximal to the NTS form a nucleus that acts as the ‘gatekeeper’ of the hypoxic ventilatory response.

If this nucleus does indeed exist, then why has it not been located by the extensive efforts of so many specialists in the field? Perhaps we are dealing with an interdependent circuit mechanism, with multiple points of signal integration? When it comes down to hand waving, either a single node or multi-nodal system of signal integration appears plausible, i.e. there may be no discrete nucleus to find. In this context and in light of all things above, we need now consider why:

1. The degree of block by *AMPK* deletion of the hypoxic ventilatory response is increased in a manner directly related to the severity of hypoxia [4].
2. The hypoxic ventilatory response can be triggered by central nervous system hypoxia alone, providing there is continued receipt of basal (normoxic) afferent input from the carotid bodies [136].
3. The hypoxic ventilatory response may be blocked by interference at any point within this circuit, e.g. carotid body resection [114] or *AMPK* deletion [4].

**Figure 4:**
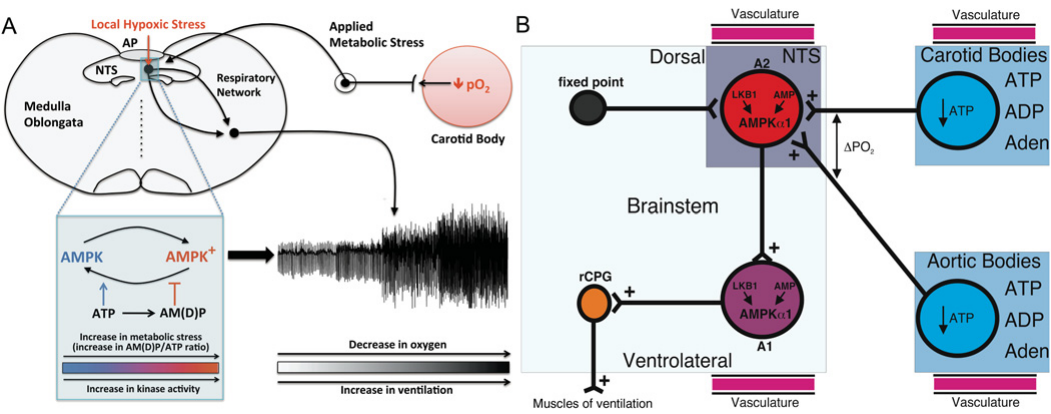
Schematic description of the new hypothesis on the integration by AMPK of local and applied metabolic stresses (**A**) Minimal model describes a single node for integration of local and applied metabolic stress by AMPK. (**B**) Extended model describes the possibility that there is capacity for signal integration, of local and applied metabolic stress, at multiple nodes within the hypoxia-responsive respiratory network. Adenosine (Aden).

We propose (Figure 4) that LKB1/AMPK signalling pathways support coincidence detection and thus signal integration at either a single node or multiple nodes within and thus activation of a hypoxia-responsive circuit that encompasses, at the very least, C2/A2 neurons within the NTS and ventrolateral A1 neurons, due to the capacity for AMPK activation by increases in the AM(D)P/ATP ratio and LKB1 [3] that may be determined by ‘local hypoxic stress’ (decreased ATP supply) and in a manner that is coupled to ‘applied metabolic stress’ (increased ATP usage) delivered via afferent inputs from peripheral chemoreceptors to the NTS, and, in turn, to ventrolateral A1 neurons and perhaps also to downstream aspects of the cardiorespiratory network. Afferent input and brainstem hypoxia could thereby determine, each in part, the set-point about which AMPK and thus the brainstem respiratory network are activated during hypoxia. Thereafter AMPK-dependent modulation of cellular metabolism [3], ion channels and thus neuronal firing frequency [21], and/or transmitter release [130,131] may facilitate efferent output and thereby deliver increased drive to breathe, in a manner that may be attenuated or augmented by appropriate regulation of AMPK expression.

In essence then, our proposal is that the LKB1/AMPK signalling pathway monitors changes in adenylate charge centrally as an index of local hypoxic stress and integrates with this applied metabolic stresses delivered by afferent chemosensory inputs, which are in turn providing an index of peripheral hypoxic (metabolic) status. If so, then perhaps we can garner more from our considerations on the regulation of afferent output from the peripheral chemoreceptors, namely the carotid and aortic bodies, in terms of their role in monitoring changes in adenylate charge and thus in the provision of an index of peripheral hypoxic stress.

As discussed in detail previously, hypoxia depolarizes type I cells through inhibition of TASK1/3 K^+^ channels, leading to voltage-gated Ca^2+^ entry, exocytosis and ultimately ATP release. Subsequently ATP stimulates postsynaptic P2X_2/3_ receptors on afferent (petrosal) nerve terminals causing excitation, but at the same time activates P2Y_2_ receptors on adjacent glial-like type II cells [77,137]. P2Y_2_ receptor activation then triggers further ATP release from type II cells into the synaptic cleft, where ATP (from both type II and type I cells) is broken down by extracellular 5′-ectonucleotidase into adenosine, which primarily activates adenosine A_2A_ receptors on type I cells [77]. Activation of A_2A_ receptors leads to further inhibition of TASK1/3 channels and enhanced type I cell depolarization [138], and further augments ATP release during hypoxia [77]; a similar system probably operates at the aortic bodies (C. Nurse, personal communication). It seems quite possible, therefore, that LKB1 may govern a set-point for metabolic homoeostasis about which both carotid and aortic bodies monitor adenylate charge as an index of hypoxic stress, by integrative inhibition of TASK1/3 channels consequent to deficits in mitochondrial ATP production that are allied to purinergic cross-talk between type I and type II cells. Via their tripartite synapse with afferent petrosal neurons [77], type I and type II cells may therefore act in concert to relay information on changes in the ‘peripheral adenylate pool’ (ATP, ADP, AMP and adenosine) to the brainstem. During the transit of re-oxygenated blood from the heart to the brainstem, the NTS may thereby co-ordinate the integration of information on adenylate charge, as an index of arterial oxygen saturation, via at least four separate and highly vascularized nodes, namely the aortic and carotid bodies, the AP/NTS and the ventrolateral medulla, in order to appropriately co-ordinate cardiorespiratory function (Figure 4).

## IN ELLIPTICAL ORBIT–AMPK AND THE REGULATION OF BLOOD FLOW AND GASEOUS EXCHANGE

Hypoxia without hypercapnia induces pulmonary vasoconstriction, and thus assists ventilation–perfusion matching by diverting blood from oxygen-deprived to oxygen-rich areas of the lung [139,140]. By contrast, systemic arteries dilate in response to tissue hypoxemia, in order to match local perfusion to local metabolism [141]. Whichever we consider, it is now evident that AMPK may be key to the regulation of vascular reactivity during metabolic stress [23,142] and may thus facilitate gaseous exchange across the body.

### AMPK and ventilation–perfusion matching at the lung

Quite unlike the hypoxic ventilatory response, hypoxic pulmonary vasoconstriction is governed locally and is mediated by mechanisms intrinsic to pulmonary arterial smooth muscles and endothelial cells. This is evident from the fact that neither central nor local regulation of the autonomic nervous system contributes to hypoxic pulmonary vasoconstriction [143–145], which remains unaffected following denervation in humans [146]. However, here too the nature of the principal signalling pathway(s) involved remains open to debate [103], although it is clear that this response relies on the modulation by hypoxia of mitochondrial metabolism [48]; pulmonary arterial smooth muscle cells depleted, by ethidium bromide, of mitochondrial DNA and thus of functional mitochondria do not respond to hypoxia [147], although inhibitors of mitochondrial oxidative phosphorylation either mimic or occlude hypoxic pulmonary vasoconstriction [148,149]. As mentioned previously and consistent with findings on carotid body type I cells [55], COX4I2 is constitutively expressed by pulmonary arterial myocytes [59] and may also act here to limit mitochondrial oxygen consumption and ROS production during hypoxia and confer, in part, the capacity of these cells to monitor oxygen supply [55].

In the light of the evidence in support of a role for mitochondria in hypoxia-response coupling, it was therefore proposed that the LKB1/AMPK signalling pathway might couple inhibition by hypoxia of mitochondrial metabolism to hypoxic pulmonary vasoconstriction [1,23,150]. Consistent with this view, AMPK-α1 activity was found to be greater in pulmonary than systemic (mesenteric) arterial smooth muscles [23] and this may in its own right afford a degree of pulmonary selectivity in terms of the capacity and nature of the response to physiological levels of hypoxia, over and above that which might be conferred by COX4I2 expression. Indeed exposure of pulmonary arterial smooth muscle to hypoxia (15–20 mmHg oxygen) precipitated increases in the AMP/ATP ratio, marked activation of AMPK and phosphorylation of acetyl-CoA carboxylase [23]; which may go some way to explain why cellular ATP levels remain remarkably stable in the presence of hypoxia [148]. Inhibition of mitochondrial oxidative phosphorylation by phenformin [151] and AICAR [66] precipitated AMPK activation and acetyl-CoA carboxylase phosphorylation within pulmonary arterial myocytes [23]. Regardless of their respective mechanism of action, hypoxia, phenformin and AICAR also induced an increase in the intracellular calcium concentration in and contraction of acutely isolated pulmonary arterial myocytes, and did so by mobilizing sarcoplasmic reticulum stores via ryanodine receptors. Most significantly AICAR evoked a sustained and reversible constriction of pulmonary artery rings, which exhibited characteristics strikingly similar to hypoxic pulmonary vasoconstriction; not least clearly defined contributions from both smooth muscles and the endothelium. Furthermore, hypoxic pulmonary vasoconstriction was inhibited by compound C [152].

In this instance it would appear that the pharmacology held true, for our most recent studies on knockout mice suggest that LKB1 and AMPK, but not CaMKK-β, are indeed required for hypoxic pulmonary vasoconstriction [153] and that dysfunction within the AMPK signalling pathway may precipitate pulmonary hypertension. Further support for this view has recently been provided by our demonstration that upon inhibition of mitochondrial oxidative phosphorylation, AMPK directly phosphorylates K_v_1.5 channels, and inhibits K^+^ currents carried by Kv1.5 in pulmonary arterial myocytes [24]. This is evident from the fact that down-regulation of K_v_1.5 expression and activity is a hallmark not only of hypoxic pulmonary vasoconstriction but also of pulmonary hypertension [154–162], and may contribute to increased survival of smooth muscle cells due to attenuation of K^+^ channel-dependent apoptosis [163–165] and also facilitate the phenotypic switch from a contractile to a proliferative state [166,167].

Consistent with the above, Zhou and co-workers have suggested that AMPK activation promotes survival of pulmonary arterial myocytes during hypoxia and thus cell proliferation by a dual mechanism, incorporating activation of autophagy by AMPK-α1 and reductions in cell death conferred by AMPK-α2 acting to reduce apoptosis via different pathways [168]. Contrary to this latter proposal, however, up-regulation of mTORC2 signalling has been proposed to underpin smooth muscle proliferation and the progression of both idiopathic and hypoxic pulmonary arterial hypertension [169], by promoting smooth muscle cell survival in a manner, at least in part, dependent on down-regulation of AMPK and consequent activation of mammalian target of rapamycin complex 1 (mTORC1). One possible explanation for these contrary prepositions could be that AMPK action is context-dependent and/or that the progression of pulmonary hypertension at different stages is governed by temporal fluctuations in AMPK activity.

### Regulation of utero-placental blood flow during hypoxia

AMPK has most recently been implicated in the regulation of uterine artery reactivity during hypoxia [170]. AMPK may, therefore, link maternal metabolic and cardiovascular responses during pregnancy and govern oxygen and nutrient supply to the foetus, thus determining foetal growth. Consistent with this view, *PRKAA1* variants most common to Andeans are positively associated with birth weight, uterine artery diameter and to alterations in the expression of genes in the mammalian target of rapamycin (mTOR) pathway that have been previously implicated in altitude-associated foetal growth restriction [2].

## CONCLUSION

In summary a growing body of evidence now supports the proposal that AMPK is key to oxygen and thus energy (ATP) supply to the body as a whole, through its contribution to the governance of the hypoxic ventilatory response, ventilation–perfusion matching at the lung and local regulation of blood and thus oxygen supply to the body systems. Aberrant AMPK expression or activity may therefore compromise system responses to hypoxia or other metabolic stressors and precipitate, for example, pulmonary hypertension [171], sleep-disordered breathing [172], hypertension [173] or foetal growth restriction [170], which are associated with either ascent to altitude [2,30] and/or metabolic syndrome-related disorders [172,174,175]. Therefore, further investigations on the role of AMPK in the regulation of ventilatory and vascular function in health and disease are warranted, in order that we may identify new therapeutic strategies allied to our growing understanding of the potential for development of subunit-selective small-molecule regulators of AMPK [73,176–178].

## Abbreviations

AICAR: 5-amino-4-imidazolecarboxamide riboside
AMPK: AMP-activated protein kinase
AP: area postrema
COX: cytochrome *c* oxidase
CaMKK-β: Ca^2+^–calmodulin-activated kinase kinase-β
LKB1: liver kinase B1
NTS: nucleus tractus solitarius
Olfr78: olfactory receptor 78
rCPG: respiratory central pattern generator
ROS: reactive oxygen species
